# The Impact of Short-Term Drought on the Photosynthetic Characteristics and Yield of Peanuts Grown in Saline Alkali Soil

**DOI:** 10.3390/plants13202920

**Published:** 2024-10-18

**Authors:** Kang He, Yang Xu, Hong Ding, Qing Guo, Dunwei Ci, Jialei Zhang, Feifei Qin, Manlin Xu, Guanchu Zhang

**Affiliations:** 1 Shandong Peanut Research Institute, Qingdao 266100, China; sdauhk@163.com (K.H.); xy52120092661@163.com (Y.X.); dingpeanut@163.com (H.D.); jone007@126.com (Q.G.); cdw_2007@126.com (D.C.); jialing_300@163.com (F.Q.); xumanlin@126.com (M.X.); 2 Shandong Academy of Agricultural Sciences, Jinan 250100, China; zhanglei19@126.com

**Keywords:** peanuts, tolerance, saline soil, interaction, drought

## Abstract

Peanuts grown in saline alkali soil are also subjected to drought stress caused by water scarcity. Therefore, we used HY25 (peanut variety) as an experimental material to investigate the effects of drought on the height of peanut main stems, length of the first lateral branch, leaf area per plant, SPAD value, net photosynthetic rate, and accumulation and distribution of photosynthetic products in saline alkali soil. The results showed that the combined stress of short-term drought and salt significantly reduced the main stem height, first lateral branch length, single plant leaf area, SPAD value, net photosynthetic rate (Pn), intercellular carbon dioxide concentration (Ci), and dry matter accumulation of peanuts, including a decrease in single plant pod yield, 100-pod weight, 100-kernel weight, and peanut yield. And the impact of drought stress on peanut yield varies at different growth stages. For example, under drought stress alone, the sensitive period is the 40th day after planting (40D) > 60th day after planting (60D) > 30th day after planting (30D). Short-term drought has the greatest impact on peanut yield at 40D, while in contrast, resuming watering after drought at 30D results in a slight but not significant increase in peanut yield in comparison with the control. Under the combined stress of drought and salt, the sensitive period of peanuts was 40D > 30D > 60D, and the single pod weight of peanuts was significantly reduced by 15.26% to 57.60% from the flowering stage to the pod stage under drought treatment compared to salt treatment, indicating a significant interaction between drought and salt stress, reducing the single leaf area and net photosynthetic rate of peanut leaves, ultimately leading to a decrease in peanut yield. Therefore, when planting peanuts in saline alkali soil, drought should be avoided, especially early drought, in order to prevent the combined effects of drought and salt stress from harming peanut yield.

## 1. Introduction

In the development process of world agriculture, crops are prone to biotic or abiotic stresses, among which salt stress is one of the main abiotic stresses. According to statistics, about 20% of the world’s land and nearly half of irrigated land are affected by salt stress. With the continuous aggravation of salinization, 30% of arable land will be affected by salt stress in the next 25 years, and this will reach 50% by 2050 [1]. Therefore, exploring high-yield and efficient cultivation techniques for crop planting under salt stress is becoming increasingly urgent. *Arachis hypogaea* L. (peanut) is recognized by the Food and Agriculture Organization of the United Nations (FAO) as a moderately salt-tolerant and suitable crop for the development and utilization of saline alkali land. In the development and utilization of saline alkali soil, the input of artificial auxiliary energy suppresses the phenomenon of niche overlap [2], thereby fully utilizing sunlight, temperature, and water to produce more products to meet human needs [3]. However, delayed germination, reduced absorption of K^+^ and Ca^2+^ [4], decreased photosynthetic rate [5] and decreased photosynthetic products [6,7] still limit crop production under salt stress [6]. Meanwhile, during the growth and development process of peanuts, they not only suffer from salt stress but are also susceptible to drought stress. Drought stress seriously affects plant yield, as water scarcity inhibits most biochemical reactions within the plant, thereby reducing its net photosynthetic rate and dry matter accumulation. Similarly, under salt stress, the energy supply for nutritional and reproductive growth in plants decreases, while the energy consumption for resisting salt stress increases, thereby affecting the maturation process of plant seeds and finally affecting crop yield and quality [8,9,10]. The high salinity of saline alkali land usually leads to a decrease in biodiversity and intensified surface evaporation, resulting in secondary drought stress and further inhibition of plant growth [11,12]. Usually, plants resist salt and drought stress by reducing water evaporation and accumulating osmoregulatory substances, such as accumulating potassium ion content, in order to maintain osmotic pressure. On the other hand, salt and drought stress can lead to secondary oxidative stress, which can also inhibit plant growth, photosynthesis rate, etc. [12]. To alleviate oxidative stress, plants typically regulate the activity of peroxidase to eliminate excess free radicals and maintain growth [13,14]. However, there are still few reports on the growth and development of peanuts under the combined treatment of drought and salt stress. Therefore, this study explored the effects of both short-term drought and salt stress at different stages on the net photosynthetic rate, dry matter accumulation, and yield of peanuts and identified the water-sensitive period for peanut growth, thereby improving peanut yield on saline alkali land.

## 2. Results

### 2.1. The Impact of Drought at Different Stages on the Agronomic Traits of Peanuts

**Figure 1:**
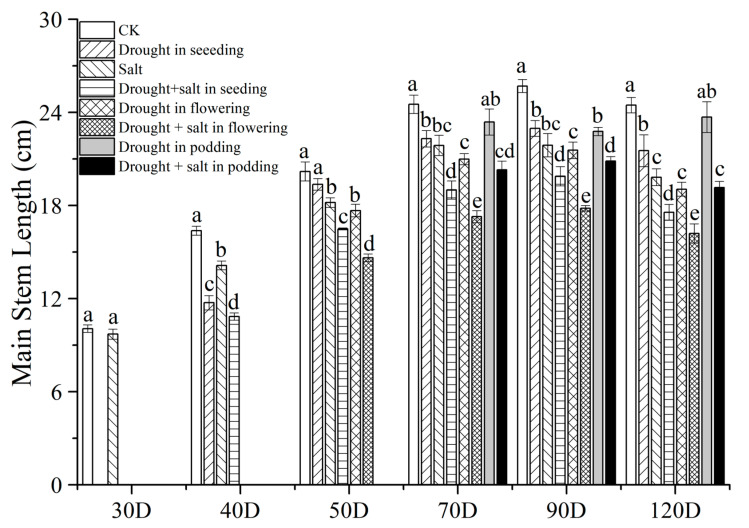
Effects of drought and salt stress on peanut stem length. CK means without treatment, Salt means salt treatment. Drought means drought. Drought + salt represents two types of stress treated together at different stages. The *X* axis represents the number of planting days. Different lowercase letters mean significant differences at the 0.05 level, and data are expressed as mean ± standard deviation (n = 3).

Salt stress significantly inhibited the main stem height of peanuts, and S treatment reduced it by 3.48% compared to CK (Figure 1). Short-term drought treatment (40D) intensified the inhibition of peanut stem height growth, with SDS treatment significantly reducing peanut stem height by 33.74% compared to CK. Similarly, at 50D, FDS (drought and salt treatment during the flowering period) treatment significantly reduced peanut stem height by 27.55% and 19.59% compared to CK and S (salt) treatments, respectively. However, at 70D, PD (drought treatment during the podding stage) treatment decreased peanut stem height by 2.94% compared to CK, and the difference was not significant; PDS (drought and salt treatment during the podding stage) treatment decreased peanut stem height by 17.21% and 7.18% compared to CK and S treatments, respectively, with significant differences. At 90D, the main stem height of CK and PD treatments was the highest, followed by SD (drought treatment during the seeding stage) and FD (drought treatment during the flowering stage), followed by S, PDS, SDS, and FDS. At 120D, the main stem height between treatments was in the order of CK > PD > SD > S > FD > PDS > SDS > FDS. Overall, the combination of early drought and salt stress (40D) had the strongest inhibitory effect on the height of peanut main stems, while the drought treatment during the flowering period had a smaller impact on peanuts.

**Figure 2:**
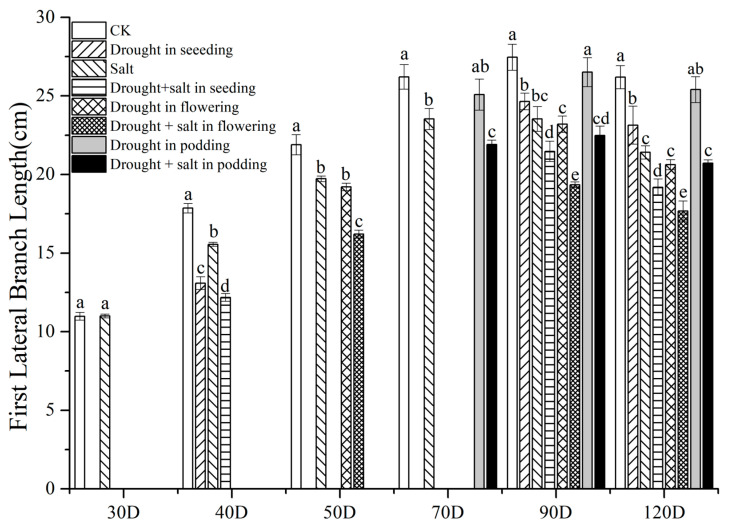
Effects of drought and salt stress on peanut first lateral branch length. CK means without treatment, Salt means salt treatment. Drought means drought. Drought + salt represents two types of stress treated together at different stages. The *X* axis represents the number of planting days. Different lowercase letters mean significant differences at the 0.05 level; data are expressed as mean ± standard deviation (n = 3).

Throughout the entire growth period, both single and compound stresses reduced the length of the first lateral branch of peanuts (Figure 2). Among them, at 30D, S treatment decreased it by 0.18% compared to CK. At 40D, SD treatment decreased it by 20.79% compared to CK, and SDS treatment decreased it by 21.72% compared to S treatment, with significant differences. At 50D, the first lateral branch length of FDS treatment was significantly reduced by 25.95% and 17.84% compared to CK and S treatments, respectively. At 70D, PD treatment decreased the first lateral branch length by 4.31% compared to CK, with no significant difference. PDS treatment decreased it by 14.18% compared to S treatment, with a significant difference. At 90D, the changes in the length of the first lateral branch between treatments were as follows: CK > PD > SD > S > FD > SDS > PDS > FDS. Short-term drought on the 40th day after sowing under salt stress had the strongest inhibitory effect on the growth of the first lateral branch of peanuts.

**Figure 3:**
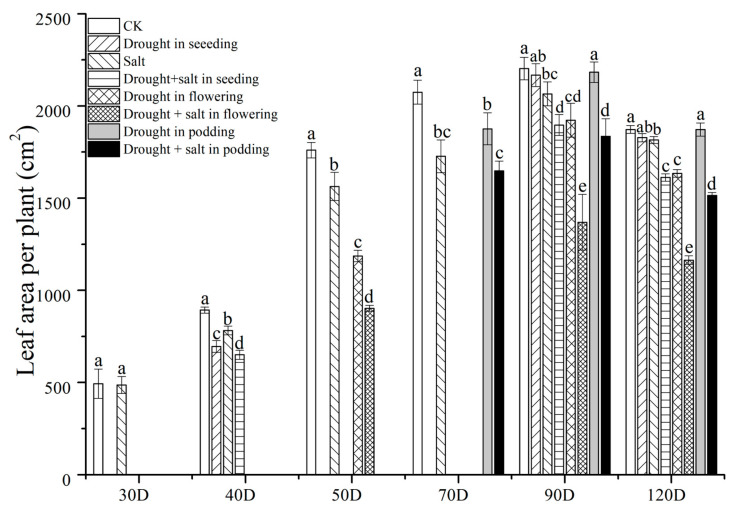
Effects of drought and salt stress on peanut single plant leaf area. CK means without treatment, Salt means salt treatment. Drought means drought. Drought + salt represents two types of stress treated together at different stages. The leaf area was calculated using a punch with a diameter of one centimeter. The *X* axis represents the number of planting days. Different lowercase letters mean significant differences at the 0.05 level; data are expressed as mean ± standard deviation (n = 3).

Drought and salt stress also reduced the leaf area per peanut plant (Figure 3). At 30D, the S treatment decreased it by 13.94% compared to the CK treatment. At 40D, SD, S, and SDS treatments decreased it by 22.08%, 12.39%, and 27.13% compared to CK, respectively. SDS treatment had the largest reduction in single plant leaf area. At 50D, there was a significant difference in single plant leaf area among CK, FD, and FDS treatments. At 90D, the size of the peanut leaf area per plant was in the order of CK > PD > SD > S > FD > SDS > PDS > FDS. Under salt stress, short-term drought on the 40th day after sowing had the strongest inhibitory effect on the peanut leaf area per plant, with the peanut leaf area per plant of the FDS treatment reducing by 37.85% and 33.72% compared to CK and S, respectively. At 120D, the differences between treatments were consistent with those at 90D, and the FDS treatment had the strongest inhibitory effect on the leaf area of peanut plants.

### 2.2. Effects of Drought at Different Stages on Photosynthetic Characteristics and SPAD Values of Peanut Leaves

The Pn of peanuts is also affected by drought and salt stress (Table 1). At 35D, both drought and salt stress reduced the Pn. SD, S, and SDS treatments decreased it by 41.45%, 19.03%, and 54.19% compared to CK, respectively. SDS treatment had the lowest Pn. At 45D, drought reduced the Pn of peanut leaves, in the order of CK > S > FD > FDS, with significant differences between treatments. At 85D, the order of Pn between treatments was CK > SD > FD > PD > SDS > S > PDS > FDS. The Pn of FDS treatment was lower than that of SDS and PDS. Under salt stress, short-term drought on the 40th day after sowing had the strongest inhibitory effect on Pn. At 115D, there was a significant interaction between short-term drought on the 40th day after sowing and short-term drought on the 60th day after sowing and salt stress on Pn. The interaction between drought and salt stress reduced Pn in peanut leaves, with the order of CK > SD > S > FD > PD > SDS > PDS > FDS.

Throughout the entire growth period, drought and salt stress reduced the Ci (intercellular carbon dioxide concentration) of peanut leaves. At 30D, S treatment significantly decreased it by 13.38% compared to CK. At 35D, SD treatment increased it by 13.3% compared to CK, while SDS treatment decreased it by 5.06% and 1.83% compared to CK and S treatments, respectively. Under no salt stress, short-term drought on the 40th day after sowing increased the Ci of peanut leaves, while short-term drought on the 40th day after sowing under salt stress reduced the Ci of peanut leaves. At 45D, drought increased the Ci of leaves; FD increased it by 32.61% compared to CK, and FDS increased it by 26.34% compared to S, with significant differences. At 65D, the order of Ci between treatments was PDS > CK > PD > S. On the 60th day after sowing, the interaction between short-term drought and salt stress increased the Ci of leaves. At 85D, the Ci between treatments was in the order of CK > S > SD > FD > PD > FDS > SDS > PDS. At 115D, the change in Ci between treatments was SDS > CK > S > FD > PD > SD > FDS > PDS.

Except for at 115D, salt stress reduced Gs (stomatal conductance) in peanut leaves during the remaining growth period. At 30D, the Gs of S-treated leaves decreased by 30.54% compared to CK, with a significant difference. At 35D, S, SD, and SDS treatments decreased Gs by 2.29%, 39.27%, and 42.65% compared to CK, respectively. On the 30th day after sowing, short-term drought had a stronger inhibitory effect on Gs in peanut leaves than salt stress. At 45D, the changes in Gs between treatments were in the order of CK > S > FDS > FD, with no significant difference between FD and FDS treatments, but significant differences between other treatments. At 65D, the change in Gs between treatments was CK > S > PD > PDS, with significant differences between treatments. At 85D, the order of Gs changes was SD > SDS > FD > CK > S > PD > FDS > PDS.

The variation in Tr (transpiration rate) in leaves between treatments varies with different treatment times. At 35D, the order of Tr variation was SDS > CK > S > SD. At 45D, the order was S > CK > FD > FDS. At 65D, the order was CK > S > PD > PDS. At 115D, the Tr of leaves treated with SDS, FDS, and PDS was the lowest, decreased by 38.71%, 34.59%, and 20.79% compared to CK, respectively, with significant differences.

Throughout the entire growth period, salt stress reduced the SPAD value of peanut leaves, and there was a significant difference between S treatment and CK. At 35D, drought increased the SPAD value of leaves, with that of SD treatment being 42.77% higher than CK and that of SDS treatment being 26.56% higher than S treatment. Drought increased the SPAD value of peanut leaves. At 45D and 65D, the changes between treatments were consistent with those at 35D. Drought increased the SPAD value of leaves, while drought increased the SPAD value of salt-stressed peanut leaves at 45D and 65D. At 85D, the changes in SPAD values between treatments were as follows: CK > SD > FD > PD > SDS > S > FDS > PDS. After rehydration, the effect of drought on SPAD values of leaves at different growth stages was relieved. There was a significant interaction between short-term drought on the 30th day after sowing and short-term drought and salt stress on the 40th day after sowing. The interaction between drought and salt stress exacerbated the harm of SDS and FDS treatments to peanuts, with SPAD values decreasing by 23.41% and 30.89% compared to CK, respectively. At 105D, the SPAD values of all treatments decreased compared to 85D, and the difference in leaf SPAD values between treatments was consistent with 85D.

### 2.3. The Impact of Drought at Different Stages on the Accumulation and Distribution of Dry Matter in Peanuts

Drought and salt stress also directly reduced the accumulation of dry matter in peanuts (Table 2). At 30D, the dry weight of leaves, stems, and roots treated with S decreased by 35.71%, 32.89%, and 38.46% compared to CK, respectively, with significant differences between treatments. There are differences in dry matter distribution due to different treatments. S treatment increased the proportion of dry matter distribution in stems but decreased the proportion of dry matter distribution in leaves and roots. At 40D, drought reduced the accumulation of dry matter in various organs of peanuts. The dry weight of leaves, stems, and roots in SD treatment decreased by 40%, 30.14%, and 25.81% compared to CK, respectively. The differences between treatments were significant. On the 30th day after sowing, short-term drought increased the proportion of dry matter distribution in stems and roots and decreased the proportion of dry matter distribution in leaves. Drought and salt stress intensified the inhibitory effect on peanut dry matter accumulation. The dry matter accumulation in leaves, stems, and roots of SDS treatment decreased by 65.81%, 60.27%, and 58.06% compared to CK and decreased by 44.04%, 44.23%, and 38.10% compared to S treatment, respectively, with significant differences between treatments. The dry weight of leaves, stems, and roots treated with FDS decreased by 69.73%, 69.84%, and 73.24% compared to CK and by 39.13%, 41.27%, and 52.5% compared to S, respectively. The differences between the treatments were significant. At the same time, FDS treatment increased the dry matter distribution ratio of plant leaves and stems compared to CK and decreased the dry matter distribution ratio of roots (50D). At 70D, the dry matter accumulation in each organ of S and PDS treatments was significantly different from CK. At 90D, the total accumulation of dry matter in each treatment plant was in the order of SD > CK > PD > S > FD > PDS > SDS > FDS.

Overall, the short-term drought treatment (FDS) on the 40th day after sowing had the most severe inhibition on peanut growth under salt stress and had the most severe impact on peanut yield. Except for FDS treatment, all treatments increased the proportion of dry matter distribution in pods compared to CK. At 120D, the trend of dry matter accumulation in various organs was consistent with that at 90D, with the lowest amount observed in FDS treatment and the second highest amount observed in SDS treatment.

### 2.4. The Impact of Drought at Different Stages on Peanut Yield

Under salt stress or no salt stress, peanuts are most sensitive to short-term drought on the 40th day after sowing (Table 3). Under no salt stress, the dry weight of individual pods among treatments was SD > CK > PD > FD, with FD treatment decreasing it by 32.47%, 34.28%, and 31.24% compared to CK, SD, and PD, respectively. FD treatment reduces the number of pods by 46.04%, 48.27%, and 40.87% compared to CK, SD, and PD, respectively. Short-term drought on the 40th day after sowing significantly reduces the number of pods per plant; Compared with CK, PD treatment significantly reduced the 100-kernel weight and yield, while SD and FD treatments showed no significant difference compared to CK. The short-term drought on the 60th day after sowing had a higher impact on the yield and 100-kernel weight of peanuts than on the 40th day and the 30th day after sowing. Under salt stress, the dry weight of individual pods among treatments was S > PDS > SDS > FDS. The dry weight of PDS, SDS, and FDS treatments decreased by 15.26%, 37.68%, and 57.60%, respectively, compared to S treatment, and the trends of these treatments were inconsistent with those under no salt stress. The differences between each treatment and the S treatment were significant. The trend of changes in the number of pods per plant between treatments is consistent with the trend of changes in the dry weight of pods per plant. On the 40th day after sowing, short-term drought reduced the number of pods per plant, leading to a decrease in yield. The changes in fruit weight and kernel weight between treatments were S > PDS > SDS > FDS, and the differences between each treatment and the S treatment were significant. The variation in peanut yield is S > PDS > FDS > SDS, with SDS having the lowest yield.

### 2.5. Correlations Between Yield and Other Indicators

**Figure 4:**
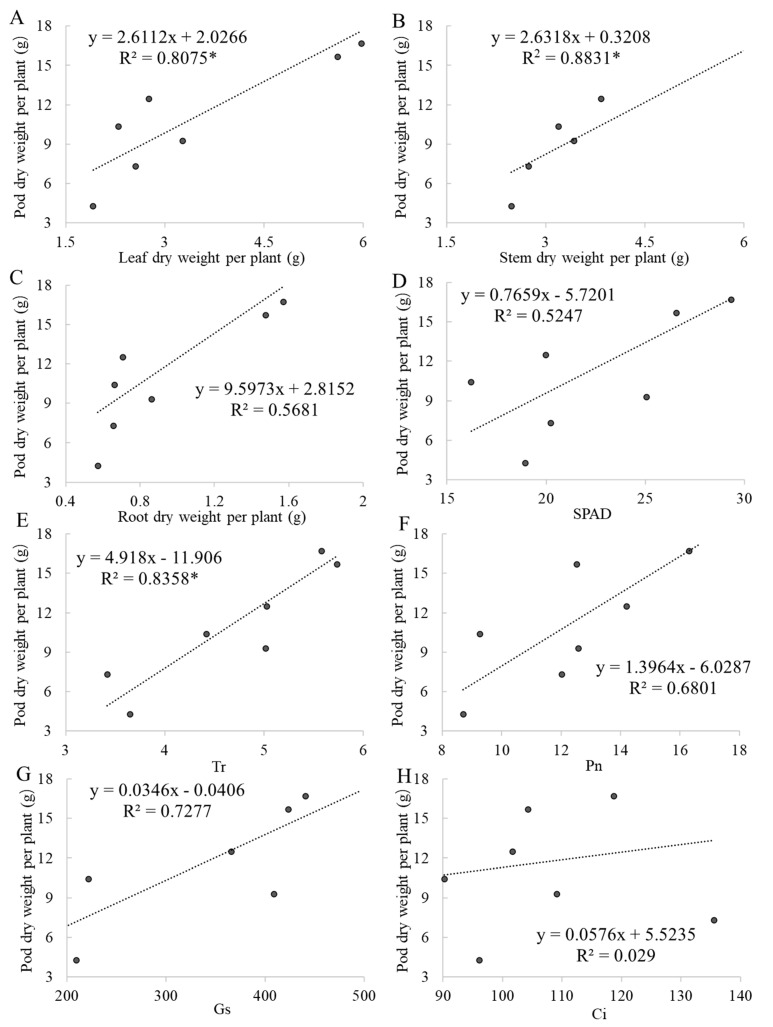
Pod dry weight per plant related to leaf dry weight per plant, stem dry weight per plant, root dry weight per plant, SPAD, Pn, Gs, Ci, Tr at harvest time. The net photosynthetic rate (Pn), intercellular carbon dioxide concentration (Ci), stomatal conductance (Gs), and transpiration rate (Tr) of leaves were measured using the CIRAS-3 portable photosynthesis system. * Indicates significant differences at *p* ≤ 0.05.

Photosynthetic indices as indicators of drought stress and the response of characteristics of peanuts under salt stress are shown in Table 3. The correlation coefficients were calculated between pod dry weight per plant and leaf dry weight per plant, stem dry weight per plant, root dry weight per plant, SPAD, and photosynthetic indices at harvest time (Figure 4). The responses of leaf dry weight per plant, stem dry weight per plant, and root dry weight per plant at harvest time were significantly related to the responses of pod yield and could be used as indicators for estimating yield.

## 3. Discussion

During the growth and development of plants, they often encounter various biotic or abiotic stresses such as diseases, pests, cold damage, freezing damage, drought, etc. Among them, drought stress is the most widespread and can cause significant crop yield reduction [15,16]. Our experimental results also showed that drought stress (SD, FD, and PD treatments) reduced the main stem height, first lateral branch length, single plant leaf area, and net photosynthetic rate of peanuts (Figure 1, Figure 2 and Figure 3, Table 1). Furlan et al. [17] found a significant decrease in peanut photosynthesis after 14 days of drought treatment, which triggered a response from the antioxidant system. Liu et al. [18] reported that drought also significantly affected the synthesis of chlorophyll and gene expression of photosynthesis in peanuts.

Compared to drought stress, plants are more sensitive and strongly responsive to salt stress [19]. Salt stress mainly inhibits plant physiological responses through ion toxicity and osmotic stress. For example, Na^+^ is a typical ion toxic stress [20], while pH can exacerbate ion toxicity. Osmotic stress mainly affects the maintenance of osmotic pressure inside and outside plant cells, leading to physiological drought and a decrease in crop yield [21]. Under the same salt stress, there are differences in salt tolerance among different genotypes of crops. At the same time, plants of the same genotype exhibit different epigenetic phenomena due to stress signal transduction and regulation of multiple metabolic pathways [22]. For example, maize affects metabolism by affecting transcription through DNA methylation, ultimately resulting in reduced root length, root surface area, and root vitality [23,24].

Salt stress not only affects the root system of plants, but also affects the photosynthetic function of plant leaves, reduces leaf stomatal conductance [9], and destroys the components of photosynthetic response proteins, thereby reducing the net photosynthetic rate [25,26,27,28]. In our experimental results, it was also shown that salt stress reduced the main stem height, first lateral branch length, single plant leaf area, SPAD value, Pn, and Gs of peanuts, ultimately leading to a decrease in dry matter accumulation and yield (Table 4).

During the process of growth and development, plants not only suffer from a single stress, but often suffer from two or more types of stress simultaneously. When facing salt stress and drought stress, plants regulate metabolism through three signaling pathways: ion and osmotic homeostasis signaling, detoxification (i.e., damage control and repair) response, and growth regulation [29], including an increase in lipid peroxidation levels [30], reducing their own water potential to maintain normal growth by increasing the concentration of alkaloids [31], or resisting stress by increasing the content of osmotic regulatory substances in the plants [32,33]. Therefore, compound stress often has a greater damaging effect than individual stress. For example, the combined effects of drought and salt stress affected the growth and development of sugar beets, leading to a decrease in yield [34], while drought and salt stress interact to reduce spinach biomass [35]. This is also consistent with our experimental results, which show that the combined stress of drought and salt significantly reduces the leaf area, leaf SPAD value, and net photosynthetic rate of peanut plants [36].

Similarly, drought treatments at different stages also had an impact on peanut yield. Under complex stress, peanut yield is the lowest and most sensitive during early drought, indicating that drought during the seedling stage has the strongest inhibitory effect on peanut growth and causes irreversible damage [37,38]. However, short-term drought treatment in the later stage did not cause a significant decrease in yield, indicating that drought in the later stage does not play a significant role in the formation of yield. For example, there are reports that drought during the flowering and needle-setting stages of peanuts can lead to significant yield reduction [36]. Our experimental results also showed that SD, FD, and PD treatments all reduced the main stem height, first lateral branch length, single plant leaf area, and net photosynthetic rate of peanuts (Figure 1, Figure 2 and Figure 3, Table 3). There are differences in the impact of drought treatment on the yield of peanuts during the harvest period at different stages, with SD > CK > PD > FD, the lowest yield being found for FD, and peanuts being most sensitive to drought at 40D. The SD yield is lower than the CK yield, which is consistent with the research results of Zhao Changxing et al. [36] and others. This may be the reason for the compensation effect after drought rehydration in the seedling stage [39,40].

The relationship between the drought tolerance index for root length density and root surface area in the deeper soil layer was positive and significant for the drought tolerance index and pod yield [41]. Our result showed that leaf dry weight, stem dry weight, and root dry weight per plant at harvest time were significantly related to the responses of pod yield, which could be used as indicators for estimating yield. The interaction of Pn between salt stress and short-term drought stress at 40D, 30D, or 60D was significant, which may be the reason that the yield in the SDS treatment was lower than that in the S treatment.

## 4. Materials and Methods

### 4.1. Experimental Design

We selected the peanut variety Huayu 25 (HY25) as the experimental material. The basic physical and chemical properties of the tested soil are shown in Table 4. The experiment was conducted in a rain shelter at Shandong Peanut Research Institute (36°81′ N, 120°50′ E). The soil was taken from the topsoil of farmland at the experimental station of Shandong Peanut Research Institute (0–20 cm). Before potting, the soil should be sieved with a sieve diameter of ≤1 cm. After mixing, it should be divided into two equal parts. One part should not be treated (referred to as CK), and the other part should be thoroughly mixed in a salt-to-dry-soil ratio of 3:1000 (referred to as S). The experiment was a randomized block experiment with a soil load of 18 kg per pot (pot diameter of 40 cm, height of 26 cm). Short-term moderate drought treatment (10 days, 45% maximum field water capacity) was conducted on the 30th day (30D), 40th day (40D), and 60th day (60D) after peanut sowing. There were a total of 8 treatments (see Table 5 for details). 30D, 40D, and 60D correspond to the seedling, flowering, and pod stages of peanut growth, respectively. Each treatment was repeated 3 times, with 6 seeds sown in each pot and 3 seedlings planted. Plants were promptly rehydrated after drought treatment and grown normally until harvest.

### 4.2. Measurement Items and Methods

#### 4.2.1. Sample Collection

Samples were taken on the 30th day (before drought treatment), 40th day (after seedling drought treatment), 50th day (after flowering drought treatment), 70th day (after pod drought treatment), 90th day (after pod drought treatment), and 120th day (after harvest) after sowing and were recorded as 30D, 40D, 50D, 60D, 90D, and 120D, respectively. At 50D, only samples treated with CK, S, FD, and FDS were collected, while at 70D, only samples treated with CK, S, PD, and PDS were collected. The main stem height, first lateral branch length, dry matter mass of each organ, and leaf area of each plant were measured, with three replicates.

We selected Huayu 25 (HY25) as the experimental material. HY25 is one of the main peanut varieties grown in our local area and also comes from our research institute, so we adopted this variety as our experimental subject. Meanwhile, this peanut variety has a certain tolerance to both salt and drought and can grow below a salt content of 0.3%.

#### 4.2.2. Determination of Net Photosynthetic Rate and SPAD Value

On the 30th day (before treatment) and at 35 days (seedling stage), 45 days (flowering stage), 65 days (pod stage), 85 days (full fruit stage), and 115 days (harvest stage) after sowing, recorded as 30D, 35D, 45D, 55D, 85D, and 115D, respectively, he net photosynthetic rate (Pn), intercellular carbon dioxide concentration (Ci), stomatal conductance (Gs), and transpiration rate (Tr) of leaves were measured using the CIRAS-3 portable photosynthesis system (PP Systems, Amesbury, USA). We selected clear weather from 9:00 to 11:00 for observation during each measurement and selected the functional leaves of each peanut plant (inverted three leaves, the third leaf facing sunlight from top to bottom), with the measured position of the leaves in the middle and upper part of the leaves, avoiding the veins; we repeated measurements 5 times for each treatment. The chlorophyll analyzer mainly measures the chlorophyll content or greenness level in leaves based on the absorption law of the chlorophyll spectrum. The SPAD value is the unit of relative chlorophyll content, which is calculated by measuring the ratio of transmitted light of leaves at two wavelengths of 650 nm and 940 nm. It is used to determine the current relative chlorophyll content in leaves. The SPAD value was measured using the SPAD-502 Chlorophyll Meter Model SPAD-502, with 5 leaves measured for each treatment and 3 replicates. At 45D, only the net photosynthetic rate and SPAD value of CK, S, FD, and FDS treatments were measured, while at 65D, only the net photosynthetic rate and SPAD value of CK, S, PD, and PDS treatments were measured [42].

#### 4.2.3. Pod Yield and Yield Composition Factors

During the harvest period, agronomic traits were evaluated, including the number of results per plant and the dry and fresh weight of pods per plant. The remaining plants were harvested uniformly, the pods were air-dried, and economically valuable pods were randomly selected to calculate the weight of 100 pods and 100 kernels and yield [43].

### 4.3. Data Analyses

Microsoft Excel 2016 was used to organize and plot the data. All data in the graphs are mean ± standard deviation (SD), and SPSS 16.0 was used for multivariate statistical analysis. One-way ANOVA (Duncan test) was used for significance difference analysis.

## 5. Conclusions

Salt stress and drought both reduced peanut yield. The interaction between drought and salt exacerbated the decline in peanut yield. Throughout the entire growth period, peanuts are more sensitive to salt and drought stress in the early stages, and salt and drought stress have a greater impact on peanut yield. It is interesting that timely restoration of watering after short-term drought does not cause a decrease in peanut yield, but salt stress can have a long-term impact on peanut yield. Therefore, when peanuts are planted on saline alkali land, early drought should be avoided by strengthening the water supply in order to prevent harm to peanuts from the dual stress of drought and salt.

## Figures and Tables

**Table 1:** Effects of drought and salt stress on photosynthetic characteristics and SPAD value in peanuts.

Growth Stage	Treatment	Pn	Ci	Gs	Tr	SPAD
		(umol·m^−2^·s^−1^)	(umol·mol^−1^)	(umol·m^−2^·s^−1^)	(mmol·m^−2^·s^−1^)	
30D	CK	16.91 ± 0.55 a	261.8 ± 5.26 a	393.34 ± 19.02 a	5.94 ± 0.19 a	46.28 ± 1.53 a
	S	13.47 ± 0.83 b	226.77 ± 10.49 b	273.2 ± 29.56 b	6.15 ± 0.15 a	39.06 ± 1.92 b
35D	CK	24.8 ± 1.15 a	240.12 ± 8.25 b	414.68 ± 12.46 a	7.05 ± 0.5 a	40.5 ± 1.73 c
	S	20.08 ± 0.42 b	232.2 ± 6.42 bc	405.18 ± 12.06 a	6.96 ± 0.18 a	36.6 ± 1.13 d
	SD	14.52 ± 1.01 c	272.06 ± 9.4 a	251.84 ± 6.45 b	5.69 ± 0.6 b	57.82 ± 3.76 a
	SDS	11.36 ± 0.62 d	227.96 ± 2.76 c	237.82 ± 11.3 b	7.1 ± 0.35 a	46.32 ± 1.81 b
45D	CK	25.82 ± 1.19 a	193.8 ± 2.17 b	466.46 ± 22.94 a	8.02 ± 0.88 b	36.48 ± 1.45 b
	S	18.8 ± 0.55 b	189 ± 4.89 b	283.15 ± 9.89 b	9.04 ± 0.63 a	33.3 ± 0.97 c
	FD	13.05 ± 1.21 c	257 ± 8.42 a	191.38 ± 2.66 c	6.2 ± 0.35 c	44.18 ± 1.36 a
	FDS	10.53 ± 1.22 d	256.6 ± 5.13 a	200.74 ± 16.76 c	5.98 ± 0.61 c	39.8 ± 2.51 b
65D	CK	16.31 ± 0.64 a	237.6 ± 10.92 a	453.46 ± 23.75 a	7.25 ± 0.88 a	42.04 ± 1.11 a
	S	15.44 ± 0.87 a	186.92 ± 4.97 b	178.81 ± 1.36 b	4.88 ± 0.38 b	32.4 ± 1.4 b
	PD	8.08 ± 1.03 b	200.93 ± 14.58 b	105.55 ± 5.47 c	3.78 ± 0.17 c	43.62 ± 2.22 a
	PDS	2.95 ± 0.42 c	251.79 ± 15.44 a	75.09 ± 4.91 d	2.83 ± 0.19 d	41.7 ± 3.07 a
85D	CK	17.72 ± 0.32 a	174 ± 6.93 a	244.19 ± 3.95 c	4.65 ± 0.13 b	43.32 ± 2.43 a
	S	10.33 ± 0.72 d	166.96 ± 1.99 b	243.49 ± 10.2 c	5.05 ± 0.64 b	31.38 ± 4.36 b
	SD	16.3 ± 2.26 a	162.02 ± 1.29 b	370.72 ± 5.26 a	6.55 ± 0.87 a	42.6 ± 3.31 a
	SDS	12.4 ± 0.45 bc	111.81 ± 2.23 d	262.37 ± 12.56 b	5.09 ± 0.43 b	33.18 ± 3.52 b
	FD	13.82 ± 0.59 b	148.28 ± 2.43 c	266.63 ± 7.16 b	5.35 ± 0.12 b	43.12 ± 1.4 a
	FDS	7.69 ± 0.19 e	140.17 ± 3.48 c	98.64 ± 1.06 e	2.79 ± 0.18 d	29.94 ± 3.47 b
	PD	11.96 ± 0.37 c	143.72 ± 3.98 c	199.77 ± 9.37 d	4.56 ± 0.09 c	42.7 ± 1.52 a
	PDS	8.75 ± 0.31 e	103.78 ± 1.07 f	76.78 ± 4.34 f	2.09 ± 0.03 e	28.64 ± 2.93 b
115D	CK	16.3 ± 0.2 a	118.7 ± 0.82 b	440.59 ± 14.6 b	5.58 ± 0.12 a	29.33 ± 2.64 a
	S	12.63 ± 0.2 c	117.7 ± 4.12 b	494.44 ± 15.56 a	5.73 ± 0.49 a	26.84 ± 2.93 a
	SD	14.2 ± 0.72 b	101.67 ± 1.15 d	365.68 ± 19.95 c	5.03 ± 0.39 a	19.98 ± 3.36 b
	SDS	12.02 ± 0.46 c	135.59 ± 1.89 a	180.19 ± 7.96 e	3.42 ± 0.23 c	20.23 ± 2.52 b
	FD	12.59 ± 0.79 c	109.11 ± 1.17 c	408.95 ± 14.42 b	5.02 ± 0.39 a	25.05 ± 2.87 ab
	FDS	8.71 ± 0.65 d	96.11 ± 2.46 e	209.63 ± 11.49 d	3.65 ± 0.1 c	18.96 ± 2.2 b
	PD	12.52 ± 0.3 c	104.33 ± 1.53 d	423.32 ± 22.86 b	5.74 ± 0.26 a	26.58 ± 1.99 a
	PDS	9.27 ± 0.4 d	90.22 ± 1.35 f	222.05 ± 11.03 d	4.42 ± 0.4 b	16.22 ± 3.49 b
Analysis of variance (*p* value)						
35D	S × D	0.038 *	<0.001 **	0.019 *	0.796	0.006 **
45D		0.022 *	0.563	0.034 *	0.024 *	0.515
65D		<0.001 **	<0.001 **	0.01 **	0.001 **	0.003 **

CK means without treatment, S means salt treatment, SD means drought treatment but no salt treatment in the seeding stage, SDS means drought and salt treatments in the seeding stage, FD means drought treatment but no salt treatment in the flowering stage, FDS means drought and salt treatments in the flowering stage, PD means drought treatment but no salt treatment in the podding stage, PDS means drought and salt treatments in the flowering stage. Different lowercase letters mean significant differences at the 0.05 level. * indicates significant differences at *p* ≤ 0.05; ** indicates significant differences at *p* ≤ 0.01; data are expressed as mean ± standard deviation (n = 3).

**Table 2:** Effects of drought and salt stress on photosynthetic material accumulation and distribution in peanuts.

		Leaf		Stem + Petiole		Root		Pod		Total
Growth Stage	Treatment	Accumulation	Distribution	Accumulation	Distribution	Accumulation	Distribution	Accumulation	Distribution	Accumulation
		(g·Plant^−1^)	Ratio (%)	(g·Plant^−1^)	Ratio (%)	(g·Plant^−1^)	Ratio (%)	(g·Plant^−1^)	Ratio (%)	(g·Plant^−1^)
30D	CK	0.84 ± 0.03 a	45.16	0.76 ± 0.025 a	40.86	0.26 ± 0.01 a	13.99	/	/	1.86 ± 0.06 a
	S	0.54 ± 0.018 b	44.63	0.51 ± 0.016 b	42.15	0.16 ± 0.005 b	13.22	/	/	1.22 ± 0.039 b
40D	CK	1.55 ± 0.05 a	46.69	1.46 ± 0.047 a	43.98	0.31 ± 0.01 a	9.34	/	/	3.31 ± 0.107 a
	S	1.02 ± 0.033 b	44.93	1.04 ± 0.034 b	45.81	0.21 ± 0.007 b	9.25	/	/	2.28 ± 0.074 b
	SD	0.93 ± 0.03 b	42.66	1.02 ± 0.033 b	46.79	0.23 ± 0.008 b	10.55	/	/	2.18 ± 0.07 b
	SDS	0.53 ± 0.017 c	42.74	0.58 ± 0.019 c	46.77	0.13 ± 0.004 c	10.48	/	/	1.24 ± 0.04 c
50D	CK	3.7 ± 0.12 a	45.74	3.68 ± 0.119 a	45.49	0.71 ± 0.023 a	8.78	/	/	8.09 ± 0.262 a
	S	1.84 ± 0.06 b	44.55	1.89 ± 0.061 b	45.76	0.4 ± 0.013 c	9.69	/	/	4.14 ± 0.134 b
	FD	1.77 ± 0.057 b	43.92	1.79 ± 0.058 b	44.42	0.47 ± 0.012 b	11.66	/	/	3.93 ± 0.127 b
	FDS	1.12 ± 0.036 c	46.26	1.11 ± 0.036 c	45.87	0.19 ± 0.006 d	7.85	/	/	2.42 ± 0.078 c
70D	CK	4.26 ± 0.138 a	37.47	4.13 ± 0.134 a	36.32	1.18 ± 0.025 a	10.38	1.8 ± 0.058 a	15.83	11.36 ± 0.286 a
	S	2.24 ± 0.073 b	32.65	3.11 ± 0.101 b	45.33	0.58 ± 0.019 b	8.45	0.93 ± 0.03 b	13.56	6.87 ± 0.222 b
	PD	4.12 ± 0.133 a	37.87	3.99 ± 0.129 a	36.67	1.11 ± 0.036 a	10.2	1.66 ± 0.054 a	15.26	10.87 ± 0.352 a
	PDS	1.97 ± 0.064 c	32.89	2.64 ± 0.086 c	44.07	0.52 ± 0.017 c	8.68	0.86 ± 0.028 c	14.36	5.99 ± 0.194 c
90D	CK	5.98 ± 0.193 a	19.32	6.71 ± 0.217 a	21.67	1.57 ± 0.051 a	5.07	16.7 ± 0.541 ab	53.94	30.96 ± 1.002 a
	S	2.76 ± 0.089 cd	13.94	3.84 ± 0.124 b	19.39	0.71 ± 0.023 e	3.59	12.49 ± 0.404 c	63.08	19.79 ± 0.641 b
	SD	5.6 ± 0.181 a	17.93	6.24 ± 0.202 a	19.99	0.99 ± 0.032 c	3.17	18.39 ± 0.595 a	58.9	31.23 ± 1.01 a
	SDS	2.56 ± 0.083 d	19.32	2.74 ± 0.089 d	20.68	0.66 ± 0.021 f	4.98	7.29 ± 0.236 e	55.02	13.24 ± 0.429 d
	FD	3.27 ± 0.106 b	19.41	3.43 ± 0.111 c	20.36	0.86 ± 0.028 d	5.1	9.29 ± 0.301 d	55.13	16.86 ± 0.545 c
	FDS	1.92 ± 0.062 f	20.78	2.48 ± 0.08 f	26.84	0.57 ± 0.049 f	6.17	4.27 ± 0.138 f	46.21	9.24 ± 0.299 e
	PD	5.62 ± 0.182 a	19.44	6.31 ± 0.204 a	21.83	1.28 ± 0.048 b	4.43	15.7 ± 0.508 b	54.31	28.9 ± 0.941 a
	PDS	2.3 ± 0.074 e	13.89	3.19 ± 0.103 c	19.27	0.66 ± 0.021 ef	3.99	10.4 ± 0.336 cd	62.84	16.55 ± 0.540 c
120D	CK	4.32 ± 0.14 a	12.58	5.99 ± 0.194 a	17.45	1.52 ± 0.049 a	4.42	22.5 ± 1.005 a	65.54	34.33 ± 1.329 a
	S	2 ± 0.065 c	8.92	3.43 ± 0.111 c	15.29	0.68 ± 0.022 e	3.03	16.32 ± 0.532 b	72.76	22.43 ± 0.729 b
	SD	3.84 ± 0.124 b	11.35	5.57 ± 0.18 b	16.46	0.95 ± 0.031 c	2.81	23.47 ± 0.766 a	69.38	33.84 ± 1.1 a
	SDS	1.75 ± 0.056 d	11.67	2.45 ± 0.079 e	16.33	0.63 ± 0.020 f	4.2	10.17 ± 0.711 d	67.8	15 ± 0.808 d
	FD	2.36 ± 0.076 c	12.83	3.06 ± 0.099 cd	16.63	0.84 ± 0.027 d	4.57	12.14 ± 0.396 c	65.98	18.4 ± 0.598 c
	FDS	1.18 ± 0.038 f	10.76	2.22 ± 0.072 f	20.24	0.65 ± 0.017 e	5.93	6.92 ± 1.213 e	63.08	10.87 ± 1.256 e
	PD	4.06 ± 0.131 ab	12.86	5.74 ± 0.186 ab	18.19	1.23 ± 0.039 b	3.9	20.53 ± 0.67 a	65.05	31.56 ± 1.026 ab
	PDS	1.26 ± 0.041 e	6.79	2.82 ± 0.091 d	15.2	0.64 ± 0.020 e	3.45	13.83 ± 0.614 c	74.56	18.55 ± 0.767 c

CK means without treatment, S means salt treatment, SD means drought treatment but no salt treatment in the seeding stage, SDS means drought and salt treatments in the seeding stage, FD means drought treatment but no salt treatment in the flowering stage, FDS means drought and salt treatments in the flowering stage, PD means drought treatment but no salt treatment in the podding stage, PDS means drought and salt treatments in the flowering stage. Different lowercase letters mean significant differences at the 0.05 level. The distribution ratio is calculated by dividing different tissues by the total weight of the peanut plant. Data are expressed as mean ± standard deviation (n = 3).

**Table 3:** Effects of drought and salt stress on peanut yield.

	100-Pod Mass (g)	100-Kernel Mass (g)	Kernel to Pod Rate (g)	Pod Mass per Plant (g)	Pod Amount per Plant
CK	158.81 ± 2.91 a	95.93 ± 1.76 ab	67.12 ± 1.23 ab	22.5 ± 1.005 a	12.35 ± 0.23 a
S	128.27 ± 1.41 b	69.05 ± 0.76 c	59.81 ± 0.86 c	16.32 ± 0.532 b	11.42 ± 0.13 b
SD	159.14 ± 1.05 a	97.89 ± 0.64 a	68.35 ± 0.45 a	23.47 ± 0.766 a	12.69 ± 0.18 a
SDS	118.67 ± 2.21 c	60.19 ± 1.12 e	56.36 ± 1.05 d	10.17 ± 0.711 d	10.85 ± 0.2 c
FD	161.56 ± 2.34 a	96.6 ± 1.36 a	68.76 ± 1.22 a	12.14 ± 0.396 c	8.34 ± 0.16 d
FDS	109.54 ± 0.99 d	56.3 ± 0.51 f	57.11 ± 0.52 d	6.92 ± 1.213 e	5.76 ± 0.05 e
PD	154.32 ± 2.44 a	93.2 ± 1.46 b	66.46 ± 1.05 b	20.53 ± 0.67 a	12.13 ± 0.19 ab
PDS	123.83 ± 2.25 c	64.27 ± 1.17 d	57.67 ± 1.05 cd	13.83 ± 0.614 c	11.12 ± 0.2 bc

CK means without treatment, S means salt treatment, SD means drought treatment but no salt treatment in the seeding stage, SDS means drought and salt treatments in the seeding stage, FD means drought treatment but no salt treatment in the flowering stage, FDS means drought and salt treatments in the flowering stage, PD means drought treatment but no salt treatment in the podding stage, PDS means drought and salt treatments in the flowering stage. Different lowercase letters mean significant differences at the 0.05 level. Data are expressed as mean ± standard deviation (n = 3).

**Table 4:** Soil physical and chemical properties.

Test Index	Organic Matter (g·kg^−1^)	pH	Total Nitrogen (g·kg^−1^)	Available P (mg·kg^−1^)	Available K (mg·kg^−1^)
Value	15.2 ± 1.2	6.7 ± 0.2	1.6 ± 0.3	45.1 ± 2.1	102.5 ± 5.3

**Table 5:** Test design.

Treatment	Non-Drought	Drought in 30D	Drought in 40D	Drought in 60D
Non-salt	CK	SD	FD	PD
Salt stress	S	SDS	FDS	PDS

## Data Availability

Data are contained within the article.

## References

[1] Wang W., Vinocur B., Altman A. (2003). Plant responses to drought, salinity and extreme temperatures: Towards genetic engineering for stress tolerance. Planta.

[2] Mason N.W.H., Bello F.D., Dole Al J.I., Lep J. (2011). Niche overlap reveals the effects of competition, disturbance and contrasting assembly processes in experimental grassland communities. J. Ecol..

[3] Fajardo A., Mcintire E.J.B. (2011). Under strong niche overlap conspecifics do not compete but help each other to survive: Facilitation at the intraspecific level. J. Ecol..

[4] Abd El-RheemKh M., Safi-Naz S. (2015). Effect of soil salinity on growth, yield and nutrient balance of peanut plants. Int. J. Chemtech. Res..

[5] Qin L., Li L., Bi C., Zhang Y., Wan S., Meng J., Meng Q., Li X. (2011). Damaging mechanisms of chilling- and salt stress to *Arachis hypogaea* L. leaves. Photosynthetica.

[6] Singh A.L., Hariprasanna K., Chaudhari V., Gor H.K., Chikani B.M. (2010). Identification of groundnut (*Arachis hypogaea* L.) cultivars tolerant of soil salinity. J. Plant Nutr..

[7] Meena H.N., Meena M., Yadav R.S. (2016). Comparative performance of seed types on yield potential of peanut (*Arachis hypogaea* L.) under saline irrigation. Field Crops Res..

[8] Shi X., Zhang Z., Dai X., Zhang G., Ci D., Ding H., Tian J. (2018). Effect of exogenous calcium application on absorption and distribution of nutrient elements in peanut under salt stress. J. Appl. Ecol..

[9] Foad M., Ismail A.M. (2007). Responses of Photosynthesis, Chlorophyll Fluorescence and ROS-Scavenging Systems to Salt Stress during Seedling and Reproductive Stages in Rice. Ann. Bot..

[10] Sakoda K., Taniyoshi K., Yamori W., Tanaka Y. (2022). Drought stress reduces crop carbon gain due to delayed photosynthetic induction under fluctuating light conditions. Physiol. Plant..

[11] Alam H., Khattak J.Z.K., Saleem M.H., Fahad S., Alkahtani J. (2020). Negative impact of long-term exposure of salinity and drought stress on native *Tetraena mandavillei* L.. Physiol. Plant..

[12] Bai X., Dai L., Sun H., Chen M., Sun Y. (2019). Effects of moderate soil salinity on osmotic adjustment and energy strategy in soybean under drought stress. Plant Physiol. Biochem..

[13] Xin P., Lü H., Gao L., Wang Y., Shao K. (2019). Dynamic responses of *Haloxylon ammodendron* to various degrees of simulated drought stress. Plant Physiol. Biochem..

[14] Muhammad I., Shalmani A., Ali M., Yang Q., Li F. (2021). Mechanisms Regulating the Dynamics of Photosynthesis Under Abiotic Stresses. Front. Plant Sci..

[15] Torres R.O., Henry A. (2018). Yield stability of selected rice breeding lines and donors across conditions of mild to moderately severe drought stress. Field Crops Res..

[16] Li C., Wang C., Zhang B., Fu R., Kaiyong G., William C. (2018). Programmed cell death in wheat (*Triticum aestivum* L.) endosperm cells is affected by drought stress. Protoplasma.

[17] Furlan A., Bianucci E., MarA D.C.T., Kleinert A., Valentine A., Castro S. (2016). Dynamic responses of photosynthesis and the antioxidant system during a drought and rehydration cycle in peanut plants. Funct. Plant Biol..

[18] Liu X., Li L., Li M., Su L., Lian S., Zhang B., Li X., Ge K., Li L. (2018). AhGLK1 affects chlorophyll biosynthesis and photosynthesis in peanut leaves during recovery from drought. Sci. Rep..

[19] Chaves M.M., Flexas J., Pinheiro C. (2009). Photosynthesis under drought and salt stress: Regulation mechanisms from whole plant to cell. Ann. Bot..

[20] Parida A., Das A. (2005). Salt tolerance and salinity effects on plants: A review. Ecotoxicol. Environ. Saf..

[21] Ehdaie B., Layne A.P., Waines J.G. (2012). Root system plasticity to drought influences grain yield in bread wheat. Euphytica.

[22] Xiong L., Schumaker K.S., Zhu J. (2002). Cell Signaling during Cold, Drought, and Salt Stress. Plant Cell.

[23] Feng K., Cui L., Lv S., Bian J., Wang M., Song W., Nie X. (2018). Comprehensive evaluating of wild and cultivated emmer wheat (*Triticum turgidum* L.) genotypes response to salt stress. Plant Growth Regul..

[24] Kumar D., Kushwaha S.K., Delvento C., Liatukas I., Vivekanand V., Svensson J.T., Henriksson T., Brazauskas G., Chawade A. (2020). Affordable Phenotyping of Winter Wheat under Field and Controlled Conditions for Drought Tolerance. Agronomy.

[25] Chen Y., Mao J., Sun L., Huang B., Ding C., Gu Y., Liao J., Hu C., Zhang Z., Yuan S. (2018). Exogenous melatonin enhances salt stress tolerance in maize seedlings by improving antioxidant and photosynthetic capacity. Physiol. Plant..

[26] Zhao F., Qin P. (2004). Protective effect of exogenous polyamines on root tonoplast function against salt stress in barley seedlings. Plant Growth Regul..

[27] Sudhir P., Murthy S.D.S. (2004). Effects of salt stress on basic processes of photosynthesis. Photosynthetica.

[28] Zhang W., Xie Z., Wang L., Li M., Lang D., Zhang X. (2017). Silicon alleviates salt and drought stress of Glycyrrhiza uralensis seedling by altering antioxidant metabolism and osmotic adjustment. J. Plant Res..

[29] Zhu J.K. (2002). Salt and drought stress signal transduction in plants. Annu. Rev. Plant Biol..

[30] Pan Y., Wu L., Yu Z. (2006). Effect of salt and drought stress on antioxidant enzymes activities and SOD isoenzymes of liquorice (*Glycyrrhiza uralensis* Fisch). Plant Growth Regul..

[31] Yahyazadeh M., Meinen R., Nsch R., Abouzeid S., Selmar D. (2018). Impact of drought and salt stress on the biosynthesis of alkaloids in *Chelidonium majus* L.. Phytochemistry.

[32] He K., Liu Q., Zhang J., Zhang G., Li G. (2023). Biochar Enhances the Resistance of *Legumes* and Soil Microbes to Extreme Short-Term Drought. Plants.

[33] Geng A., Lian W., Wang Y., Liu M., Zhang Y., Wang X., Chen G. (2024). Molecular Mechanisms and Regulatory Pathways Underlying Drought Stress Response in Rice. Int. J. Mol. Sci..

[34] Hemasundar A., Seher Y., Monika S., Bahar A.S., Muhammad A. (2023). Salt and drought stress-mitigating approaches in sugar beet (*Beta vulgaris* L.) to improve its performance and yield. Planta.

[35] Ors S., Suarez D.L. (2017). Spinach biomass yield and physiological response to interactive salinity and water stress. Agric. Water Manag..

[36] Zhao C., Cheng X., Wang Y., Wang M. (2012). Effects of drought stress on peanut growth during different growth stages and compensatory effect after water recovery. Chin. J. Oil Crop Sci..

[37] Thangthong N., Jogloy S., Pensuk V., Kesmala T., Vorasoot N. (2016). Distribution patterns of peanut roots under different durations of early season drought stress. Field Crops Res..

[38] Puangbut D., Jogloy S., Vorasoot N., Craig K. (2018). Root distribution pattern and their contribution in photosynthesis and biomass in jerusalem artichokeunder drought conditions. Pak. J. Bot..

[39] Senapati N., Stratonovitch P., Paul M.J., Semenov M.A. (2019). Drought tolerance during reproductive development is important for increasing wheat yield potential under climate change in Europe. J. Exp. Bot..

[40] Lan Y., Chawade A., Kuktaite R., Johansson E. (2022). Climate Change Impact on Wheat Performance—Effects on Vigour, Plant Traits and Yield from Early and Late Drought Stress in Diverse Lines. Int. J. Mol. Sci..

[41] Ding H., Zhang Z., Kang T., Dai L., Song W. (2017). Rooting traits of peanut genotypes differing in drought tolerance under drought stress. Int. J. Plant Prod..

[42] Zhang G., Liu Q., Zhang Z., Ci D., Zhang J., Xu Y., Guo Q., Xu M., He K. (2023). Effect of Reducing Nitrogen Fertilization and Adding Organic Fertilizer on Net Photosynthetic Rate, Root Nodules and Yield in Peanut. Plants.

[43] Ci D., Qin F., Tang Z., Zhang G., Zhang J., Si T., Yang J., Xu Y., Yu T., Xu M. (2023). Arbuscular Mycorrhizal Fungi Restored the Saline–Alkali Soil and Promoted the Growth of Peanut Roots. Plants.

